# CD56^neg^CD16^+^ NK cells are activated mature NK cells with impaired effector function during HIV-1 infection

**DOI:** 10.1186/1742-4690-10-158

**Published:** 2013-12-18

**Authors:** Jeffrey M Milush, Sandra López-Vergès, Vanessa A York, Steven G Deeks, Jeffrey N Martin, Frederick M Hecht, Lewis L Lanier, Douglas F Nixon

**Affiliations:** 1 Division of Experimental Medicine, Department of Medicine, University of California, San Francisco, CA 94110, USA; 2 Department of Microbiology and Immunology and the Cancer Research Institute, University of California, San Francisco, CA 94143, USA; 3 Positive Health Program, Department of Medicine, San Francisco General Hospital, San Francisco, CA 94110, USA; 4 Department of Epidemiology and Biostatistics, University of California, San Francisco, CA 94107, USA; 5 Department of Microbiology, Immunology & Tropical Medicine, The George Washington University, Washington DC 20037, USA; 6 Current address: Gorgas Memorial Institute for Health Studies, Ave. Justo Arosemena y Calle No. 35, Panama 0816-02593, Republic of Panama

**Keywords:** Natural killer cells, NK cells, CD7, Human immunodeficiency virus, HIV-1, HIV pathogenesis, CD56neg NK cells

## Abstract

**Background:**

A subset of CD3^neg^CD56^neg^CD16^+^ Natural Killer (NK) cells is highly expanded during chronic HIV-1 infection. The role of this subset in HIV-1 pathogenesis remains unclear. The lack of NK cell lineage-specific markers has complicated the study of minor NK cell subpopulations.

**Results:**

Using CD7 as an additional NK cell marker, we found that CD3^neg^CD56^neg^CD16^+^ cells are a heterogeneous population comprised of CD7^+^ NK cells and CD7^neg^ non-classical myeloid cells. CD7^+^CD56^neg^CD16^+^ NK cells are significantly expanded in HIV-1 infection. CD7^+^CD56^neg^CD16^+^ NK cells are mature and express KIRs, the C-type lectin-like receptors NKG2A and NKG2C, and natural cytotoxicity receptors similar to CD7^+^CD56^+^CD16^+^ NK cells. CD7^+^CD56^neg^ NK cells in healthy donors produced minimal IFNγ following K562 target cell or IL-12 plus IL-18 stimulation; however, they degranulated in response to K562 stimulation similar to CD7^+^CD56^+^ NK cells. HIV-1 infection resulted in reduced IFNγ secretion following K562 or cytokine stimulation by both NK cell subsets compared to healthy donors. Decreased granzyme B and perforin expression and increased expression of CD107a in the absence of stimulation, particularly in HIV-1-infected subjects, suggest that CD7^+^CD56^neg^CD16^+^ NK cells may have recently engaged target cells. Furthermore, CD7^+^CD56^neg^CD16^+^ NK cells have significantly increased expression of CD95, a marker of NK cell activation.

**Conclusions:**

Taken together, CD7^+^CD56^neg^CD16^+^ NK cells are activated, mature NK cells that may have recently engaged target cells.

## Background

Natural killer (NK) cells comprise 5–20% of peripheral blood mononuclear cells (PBMC) in humans and play a fundamental role in the defense against viral infections, as well as in tumor surveillance, and help shape adaptive immune responses through their production of cytokines [1-3]. NK cells are traditionally identified as CD3^neg^, CD14^neg^, CD19^neg^ lymphocytes expressing CD56 (neural cell adhesion molecule) and NKp46 [4], although not all human NK cells express NKp46 [1]. NK cells are typically characterized into two main subsets; CD56^dim^CD16^+^ NK cells, which comprise approximately 90% of circulating NK cells and are considered mature, and CD56^bright^CD16^neg/dim^ NK cells, which represent approximately 10% and are considered immature [1,5]. A diverse array of activating and inhibitory receptors controls their function. Upon activation, NK cells secrete IFNγ and other cytokines and kill susceptible target cells [2].

Initial studies of NK cells during Human Immunodeficiency Virus (HIV-1) infection found significantly reduced absolute numbers of CD3^neg^CD56^+^CD16^+^ NK cells with a concomitant increase in CD3^neg^CD56^neg^CD16^+^ cells [6-8]. In HIV-1-infected subjects, CD3^neg^CD56^neg^CD16^+^ cells are described as having decreased expression of activating receptors (i.e. NKp30 and NKp46) and increased expression of inhibitory receptors (i.e. LIR-1 and inhibitory KIR), and have poor cytolytic, proliferative, and cytokine-producing capabilities [8-14]. It has been hypothesized that the expansion of this defective CD3^neg^CD56^neg^CD16^+^ population might be one mechanism by which HIV-1 subverts the NK cell response.

Expansion of CD3^neg^CD56^neg^CD16^+^ cells has also been observed in other infectious diseases (i.e. hantavirus and chronic hepatitis C virus (HCV) infection) [15,16], as well as in ocular myasthenia gravis [17] and dermatomyositis [18]. Common to each of these pathological conditions is immune activation. Indeed, chronic immune activation is a hallmark of untreated HIV-1 disease and results in accelerated immunosenescence [19]. In healthy subjects, one study has suggested that NK cells might proliferate and die more rapidly than do T cells however this requires further investigation [20]. We recently demonstrated that CD57 is a marker of terminally differentiated NK cells [21], and that during acute cytomegalovirus (CMV) infection NKG2C^hi^-expressing NK cells acquire expression of CD57 [22]. Importantly, treatment with antiviral drugs in HIV-1 and HCV infection or immunosuppressants for myasthenia gravis and dermatomyositis decrease the frequency of CD3^neg^CD56^neg^CD16^+^ cells over the course of months to levels found in healthy subjects [11,16-18].

There remain many unanswered questions regarding the phenotype, function, and origin of CD3^neg^CD56^neg^CD16^+^ cells in healthy individuals, and how they compare to the expanded population found during chronic infectious diseases such as HIV-1 infection. We recently demonstrated that including CD7 as an additional positive NK cell marker is an effective method for studying non-classical NK cell subsets [23]. Using CD7, we expand our collective knowledge about the phenotype and function of CD56^neg^CD16^+^ cells in healthy and HIV-1-infected subjects. Based upon previous observations that chronic viremia is associated with an increased frequency of CD56^neg^CD16^+^ cells, we tested the hypothesis that CD7 would refine the population of NK cells defined as CD56^neg^CD16^+^ cells and provide evidence that CD7^+^CD56^neg^CD16^+^ NK cells are mature NK cells.

## Results

### CD56^neg^CD16^+^ cells are a mixed population of myeloid and NK cells that is expanded during chronic HIV-1 infection

**Figure 1:**
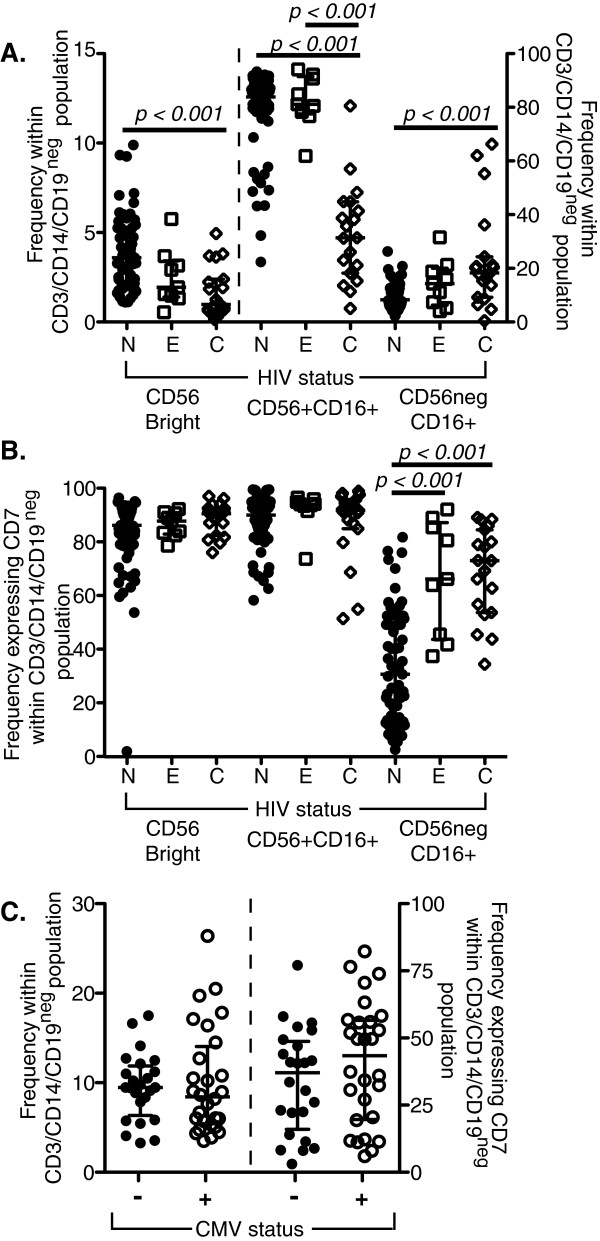
**Distribution of NK cell subsets. A**, Frequency of classically defined NK cell subsets within the CD3^neg^ CD14^neg^ CD19^neg^ population of healthy donors ((•) N, n = 63) and early- ((☐) E, n = 9) and chronically- (() C, n = 19) HIV-1-infected subjects. **B**, Frequency of CD3^neg^ CD14^neg^ CD19^neg^ cells expressing CD7 within the CD56^bright^CD16^neg^, CD56^+^CD16^+^ and CD56^neg^CD16^+^ subsets in healthy ((•) N, n = 63) and early- ((☐) E, n = 9) and chronically- (() C, n = 19) HIV-1-infected subjects. **C**, Frequency of CD56^neg^CD16^+^ cells within the CD3^neg^ CD14^neg^ CD19^neg^ population (left axis) and of CD3^neg^ CD14^neg^ CD19^neg^ cells expressing CD7 within the CD56^neg^CD16^+^ subset (right axis) of CMV-negative (closed circles) and CMV-positive (open circles) healthy donors. The median and 25th and 75th percentile are indicated on each graph.

PBMC from HIV-1-negative and HIV-1-infected subjects (Table 1) from the OPTIONS (early infection) and SCOPE (chronic infection) cohorts were gated on CD3^neg^CD14^neg^CD19^neg^ cells and the three major NK cell subsets were assessed: CD56^bright^CD16^neg^, CD56^+^CD16^+^, and CD56^neg^CD16^+^ (Additional file 1). A significant decrease in the frequency of CD56^bright^CD16^neg^ and CD56^+^CD16^+^ NK cells with a significant increase in the frequency of CD56^neg^CD16^+^ cells was observed in HIV-1-infected subjects, particularly during chronic HIV-1 infection (Figure 1A). These results are in agreement with previous studies describing CD56^neg^CD16^+^ cells as highly expanded during chronic HIV-1 (and HCV) infections [8,12,16,24].

**Table 1:** **Early and chronic HIV-1-infected patient viral loads and CD4**^**+ **^**T cell counts**

**Patient ID**	**Cohort**	**Antiretroviral status**	**Viral load**	**CD4**^+^**T cell count**
**647**	OPTIONS	None	73988	594
**683**	OPTIONS	None	95903	480
**722**	OPTIONS	None	124070	522
**730**	OPTIONS	None	2185	594
**792**	OPTIONS	None	128274	281
**804**	OPTIONS	None	4812	800
**830**	OPTIONS	None	14207	850
**858**	OPTIONS	None	56133	466
**876**	OPTIONS	None	149012	343
**1049**	SCOPE	None	336630	218
**1070**	SCOPE	None	365000	331
**1091**	SCOPE	None	59935	288
**1208**	SCOPE	None	17641	1054
**1217**	SCOPE	None	19933	281
**1311**	SCOPE	None	56566	647
**1335**	SCOPE	None	50738	501
**1538**	SCOPE	None	47025	612
**1566**	SCOPE	None	34418	853
**1571**	SCOPE	None	21734	381
**1587**	SCOPE	None	13589	469
**1588**	SCOPE	None	25552	891
**1596**	SCOPE	None	23364	621
**1597**	SCOPE	None	12593	498
**1606**	SCOPE	None	24458	678
**1654**	SCOPE	None	91597	441
**1662**	SCOPE	None	236000	584
**1679**	SCOPE	None	145465	288
**4015**	SCOPE	None	63400	714

A recent gene expression study of CD56^neg^CD16^+^ cells, traditional CD56^+^CD16^+^ NK cells, and myeloid cells found that the transcriptome of CD56^neg^CD16^+^ cells was more similar to myeloid cells than to traditional CD56^+^CD16^+^ NK cells [25]. Therefore, we hypothesized that CD56^neg^CD16^+^ cells are a mixed population of myeloid and NK cells that could be distinguished using CD7 [23]. In HIV-1-negative controls, less than 40% of CD56^neg^CD16^+^ cells co-express CD7, indicating the CD56^neg^CD16^+^ population is not a homogeneous population of NK cells (Figure 1B). During early HIV-1 infection, there is a significant expansion in the frequency of CD7^+^CD56^neg^CD16^+^ NK cells that appears to persist into chronic HIV-1 infection; however, CD56^neg^CD16^+^ cells remain a mixed myeloid and NK cell population (Figure 1B). In the absence of CD7 gating we did not observe the expansion of CD56^neg^CD16^+^ cells during early infection (Figure 1A). Persistent HIV-1 viremia appears to be important for the accumulation of CD7^+^CD56^neg^CD16^+^ NK cells [8,12,16,24]. No significant difference was observed in the frequency of CD7^+^CD56^neg^CD16^+^ NK cells in healthy, non-HIV infected individuals, including both cytomegalovirus-seropositive and cytomegalovirus-seronegative subjects (Figure 1C). Taken together, CD56^neg^CD16^+^ cells are a mixed population of cells, a subset of which are CD7^+^ NK cells that are expanded during persistent HIV-1 viremia.

### CD7^+^CD56^neg^CD16^+^ and CD7^+^CD56^+^CD16^+^ NK cells are phenotypically similar in healthy donors; however, HIV-1 infection significantly alters the phenotype of both NK cell subsets

In the absence of CD7 gating, CD56^neg^CD16^+^ cells appear to have no or very low levels of NKp30 and granzyme B expression. However, when the CD7^+^CD56^neg^CD16^+^ NK cells are gated and compared to mature CD7^+^CD56^+^CD16^+^ NK cells, little difference between the cell subsets was observed (Additional file 2). As these data are in disagreement with previous reports [12,26], we sought to further characterize CD7^+^CD56^neg^CD16^+^ NK cells compared to CD7^+^CD56^+^CD16^+^ NK cells in healthy and HIV-1-infected individuals.

**Figure 2:**
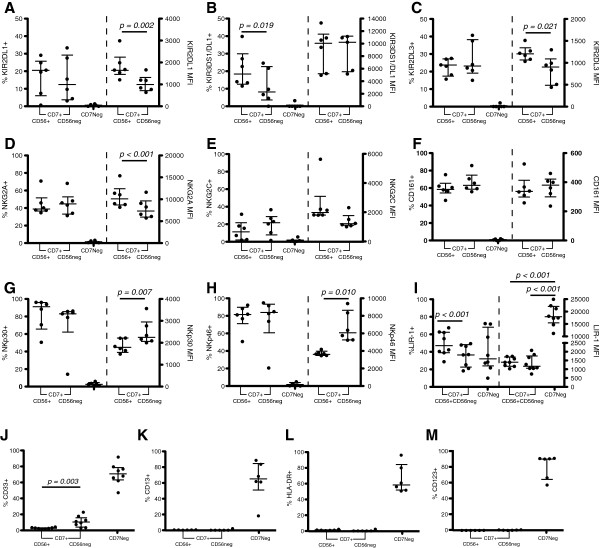
**Phenotypic characterization of CD7**^**+**^**CD56**^**+**^**CD16**^**+ **^**and CD7**^**+**^**CD56**^**neg**^**CD16**^**+ **^**NK cells and CD7**^**neg**^**CD56**^**neg**^**CD16**^**+ **^**cells in healthy donors.** CD3^neg^CD14^neg^CD19^neg^ cells were gated for CD56^+^ and CD56^neg^ cells and plotted against CD7 to identify CD7^+^ NK cells and CD7^neg^ monocyte or DC-like cells. The percentage of CD7^+^CD56^+^CD16^+^ and CD7^+^CD56^neg^CD16^+^ NK cells and CD7^neg^CD56^neg^CD16^+^ monocyte or DC-like cells expressing each receptor was determined (n = 6). **A**-**I**, Expression of NK cell-associated receptors on the different cell subsets. **J**-**M**, Expression of myeloid-associated markers on the different cell subsets. The median and 25th and 75th percentile are indicated on each graph.

We assessed the expression of Killer cell Immunoglobulin-like Receptors (KIRs), C-type lectin-like receptors (NKG2A, NKG2C and CD161), and Natural Cytotoxicity Receptors (NCRs; NKp30, and NKp46), as well as receptors typically found on myeloid cells (CD33, CD13, and HLA-DR) in HIV-1-negative donors (Figure 2). No significant difference was observed in the frequency of KIR2DL1- and KIR2DL3-bearing cells between CD7^+^CD56^+^CD16^+^ and CD7^+^CD56^neg^CD16^+^ NK cells, although CD7^+^CD56^neg^CD16^+^ NK cells had a significantly lower frequency of KIR3DS1/DL1-positive NK cells. The density of KIR2DL1 and KIR2DL3 as measured by mean fluorescent intensity (MFI) was significantly lower on CD7^+^CD56^neg^CD16^+^ NK cells, whereas no significant difference in MFI of KIR3DS1/DL1 was observed between the NK cell subsets (Figure 2A-C). With regard to the C-type lectin-like receptors, similar frequencies of both NK cell subsets expressed NKG2A and NKG2C. While both NK cell subsets had similar densities of NKG2C expression, the density of NKG2A was significantly lower on CD7^+^CD56^neg^CD16^+^ NK cells (Figure 2D-E). No significant differences were observed in the frequency of NKp30 and NKp46 between the NK cell subsets (Figure 2G-H). In contrast to previous studies, NKp30 and NKp46 were expressed at significantly greater densities on CD7^+^CD56^neg^CD16^+^ NK cells compared with CD7^+^CD56^+^CD16^+^ NK cells (Figure 2G-H). By including all CD7^+^CD56^+^ NK cells (i.e. CD56^br^CD16^neg^, CD56^br^CD16^dim^, and CD56^+^CD16^+^) in the analyses as was done by Mavilio *et al.*[12], the MFI of NKp30 and NKp46 expression remained significantly greater in CD7^+^CD56^neg^CD16^+^ NK cells compared with total CD7^+^CD56^+^ NK cells and CD7^+^CD56^+^CD16^+^ NK cells (Additional file 3). However, total CD7^+^CD56^+^ NK cells showed a trend (p = 0.070) toward greater NKp46 density compared to CD7^+^CD56^+^CD16^+^ NK cells (Additional file 3B). No significant differences were observed in the frequency or density of CD161 expression between CD7^+^CD56^+^CD16^+^ and CD7^+^CD56^neg^CD16^+^ NK cells (Figure 2F). A prior study of CD56^neg^CD16^+^ NK cells indicated a significantly greater expression of the inhibitory receptor LIR-1 on CD56^neg^CD16^+^ compared to CD56^+^CD16^+^ NK cells [12]. However, using CD7 to further delineate NK cells, we observed that LIR-1 density on CD7^+^CD56^+^CD16^+^ and CD7^+^CD56^neg^CD16^+^ NK cells was not significantly different (Figure 2I). Indeed, the apparent higher levels of LIR-1 expression on CD56^neg^CD16^+^ cells was likely due to the contamination with CD7^neg^CD56^neg^CD16^+^ myeloid cells that have five-fold greater LIR-1 density than CD7^+^ NK cells. Importantly, CD7^neg^CD56^neg^CD16^+^ cells did not express any of these NK cell-associated receptors. However, high frequencies of CD7^neg^CD56^neg^CD16^+^ cells did express CD33, CD13, and HLA-DR, markers that are classically expressed by myeloid cells (Figure 2J-L). Recently, Bigley *et al.* observed that a subset of CD123+ plasmacytoid dendritic cells express CD7 [27]. Although approximately 80% of the CD7^neg^CD56^neg^CD16^+^ cells expressed CD123, no expression of CD123 was observed on CD7^+^CD56^+^CD16^+^ or CD7^+^CD56^neg^CD16^+^ NK cells (Figure 2M). These results are in agreement with Bigley *et al.* who reported that CD7^+^ CD123^+^ plasmacytoid dendritic cells were CD16-negative [27]. The phenotypic profile of CD7^neg^CD56^neg^CD16^+^ cells is largely consistent with non-classical CD14^neg^CD16^+^ monocytes and a subset of dendritic cells (DCs) designated slanDCs [28] that fall within the lymphoid gate based on light scattering properties.

**Figure 3:**
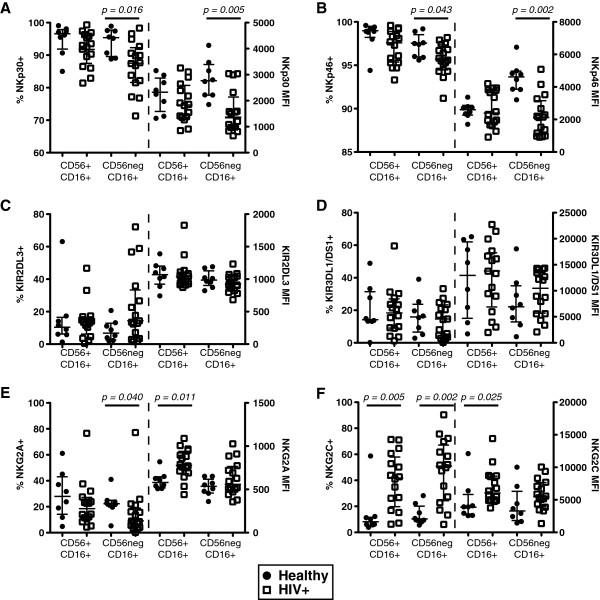
**Assessment of CD7**^**+**^**CD56**^**+**^**CD16**^**+ **^**and CD7**^**+**^**CD56**^**neg**^**CD16**^**+ **^**NK cells in healthy (n = 8) and chronically (n = 16) infected HIV-1 donors. A** and **B**, The frequency and mean fluorescence intensity (MFI) of NKp30 **(A)**, NKp46 **(B)**, KIR2DL3 **(C)**, KIR3DL1/DS1 **(D)**, NKG2A **(E)** and NKG2C **(F)** within CD7^+^CD56^neg^CD16^+^ and CD7^+^CD56^+^CD16^+^ NK cells. The median and 25th and 75th percentile are indicated on each graph.

To determine the impact of HIV-1 infection on the phenotype of CD7^+^CD56^+^CD16^+^ and CD7^+^CD56^neg^CD16^+^ NK cells, KIR, C-type lectin-like receptors, and NCRs were assessed in 16 chronically HIV-1-infected subjects and 8 healthy controls (Figure 3). HIV-1-infected subjects trended towards a reduced frequency of NKp30- and NKp46-positive CD7^+^CD56^+^CD16^+^ NK cells compared to healthy controls. However, the frequency of NKp30- and NKp46-expressing CD7^+^CD56^neg^CD16^+^ NK cells and the density of NKp30 and NKp46 expression on CD7^+^CD56^neg^CD16^+^ NK cells were significantly lower in HIV-1-infected subjects. Importantly, there were no significant differences in the frequencies of cells expressing NCRs or the density of the NCRs between CD7^+^CD56^+^CD16^+^ and CD7^+^CD56^neg^CD16^+^ NK cells in the HIV-1-infected subjects (Figure 3A and B). Assessment of KIR2DL3 and KIR3DL1/DS1 did not reveal any significant differences between subsets of NK cells or the HIV-1 infection status of the subject (Figure 3C and D). A significantly lower frequency of CD7^+^CD56^neg^CD16^+^ NK cells within HIV-1-infected subjects expressed NKG2A, while the density of NKG2A expression was significantly greater on CD7^+^CD56^+^CD16^+^ NK cells in HIV-1-infected subjects (Figure 3E). The frequency of NK cells and density of the activating receptor NKG2C is elevated in CMV-infected individuals [22,29,30], a co-infection that is highly prevalent (>98% in the SCOPE cohort [31]) in HIV-1-infected subjects. To this end, it was not unexpected to observe that the frequency of NKG2C-bearing cells was significantly greater on both subsets of NK cells in HIV-1-infected subjects compared to healthy controls (Figure 3F). Furthermore, the density of NKG2C expression was significantly greater on CD7^+^CD56^+^CD16^+^ NK cells in HIV-1-infected compared to uninfected individuals. However, no significant differences were observed between CD7^+^CD56^+^CD16^+^ and CD7^+^CD56^neg^CD16^+^ NK cell subsets within the HIV-1-infected subjects (Figure 3F). The increased frequency and density of NKG2C^+^ NK cells in HIV-1-infected subjects likely represents the higher prevalence of cytomegalovirus infection or reactivation in these subjects [31]. Taken together these results indicate that HIV-1 infection has a significant impact on the overall NK cell phenotype. However, with the exception of NKp30 and NKp46, CD7^+^CD56^neg^CD16^+^ NK cells do not appear to be significantly altered compared to CD7^+^CD56^+^CD16^+^ NK cells within HIV-1-infected subjects.

### CD7^+^CD56^neg^CD16^+^ NK cells are mature NK cells

**Figure 4:**
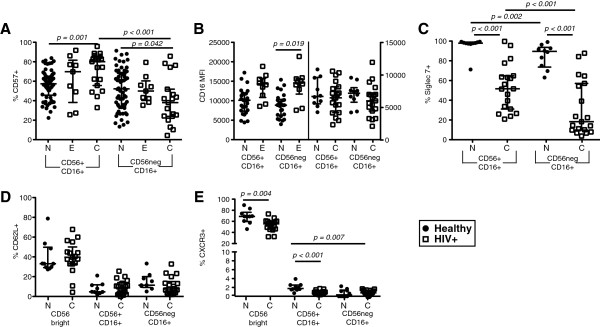
**Assessment of maturation in NK cell subsets of healthy and HIV-1-infected subjects. A**, Frequency of CD7^+^CD56^+^CD16^+^ and CD7^+^CD56^neg^CD16^+^ NK cells expressing CD57 in healthy (n = 62) and early (n = 9) and chronically (n = 19) HIV-1-infected subjects. **B**, The MFI of CD16 expression was assessed on CD7^+^CD56^+^CD16^+^ and CD7^+^CD56^neg^CD16^+^ NK cells in healthy (n = 52) and early (n = 9) HIV-1-infected subjects (left half), and in healthy (n = 10) and chronically HIV-1-infected subjects (n = 19) (right half). The healthy controls and early HIV-1 subjects were stained and analyzed in a different study with an anti-CD16 antibody conjugated to a different fluorophore than the healthy controls and chronic HIV-1-infected subjects. Therefore, early and chronic HIV-1-infected subjects cannot be directly compared with regard to mean fluorescent intensity. **C**, The frequency of CD7^+^CD56^+^CD16^+^ and CD7^+^CD56^neg^CD16^+^ NK cells expressing Siglec-7. **D-E**, The frequency of CD7^+^CD56^bright^CD16^neg^, CD7^+^CD56^+^CD16^+^ and CD7^+^CD56^neg^CD16^+^ NK cells expressing **D)** CD62L and **E)** CXCR3. The same healthy (n = 10) and chronic HIV-1-infected subjects (n = 19) used in 4B were used to assess Siglec-7, CD62L and CXCR3. The median and 25th and 75th percentile are indicated on each graph. ( = healthy;  = HIV-1-infected).

To determine whether differences in maturation existed between CD7^+^CD56^neg^CD16^+^ and CD7^+^CD56^+^CD16^+^ NK cells, we assessed the expression of CD16, CD57, Siglec-7, CD62L, and CXCR3. A significantly greater frequency of CD7^+^CD56^+^CD16^+^ NK cells in HIV-1-infected subjects expressed CD57 compared to healthy subjects. Although CD57 expression on CD7^+^CD56^+^CD16^+^ and CD7^+^CD56^neg^CD16^+^ NK cells in healthy controls was not significantly different, the frequency of CD57^+^CD7^+^CD56^neg^CD16^+^ NK cells in HIV-1-infected subjects was significantly less compared to CD57^+^CD7^+^CD56^+^CD16^+^ NK cells (~44% versus ~54% respectively; Figure 4A). When we separated healthy subjects by CMV serology status, we did not observe a significant difference in CD57 expression between the NK cell subsets (data not shown). CD16 expression increases with NK cell maturation [21]. We observed that early HIV-1 infection resulted in a significant increase in CD16 expression on both subsets of NK cells compared to healthy controls (Figure 4B left half). Because the studies were performed at different times, we were unable to directly compare the density of CD16 expression (based on MFI) in early and chronic HIV-1 infection. However, in contrast to early infection, CD16 expression did not appear to differ significantly on CD7^+^CD56^+^CD16^+^ or CD7^+^CD56^neg^CD16^+^ NK cell subsets in chronically HIV-1-infected subjects compared to healthy controls (Figure 4B, right half). Recently, HIV-1 infection was reported to induce a rapid and sustained decrease in Siglec-7 expression on NK cells, which is associated with impaired function [11]. We observed that greater than 90% of CD7^+^CD56^+^CD16^+^ NK cells in healthy donors expressed Siglec-7, whereas approximately 50% of this NK cell subset expressed Siglec-7 in HIV-1-infected subjects (Figure 4C). Furthermore, we found that a lower frequency of CD7^+^CD56^neg^CD16^+^ NK cells expressed Siglec-7 in both healthy controls and HIV-1-infected subjects, but the loss of Siglec-7 on CD7^+^CD56^neg^CD16^+^ NK cells of HIV-1-infected subjects was significantly greater (Figure 4C). During NK cell maturation from an immature CD56^bright^CD16^neg^ to a mature CD56^+^CD16^+^ phenotype, CD62L and CXCR3 expression are decreased [32-34]. In accordance with a mature phenotype, CD7^+^CD56^neg^CD16^+^ NK cells have similar CD62L expression compared to CD7^+^CD56^+^CD16^+^ NK cells, which is significantly lower than that expressed by CD7^+^CD56^bright^CD16^neg^ immature NK cells (Figure 4D). Similarly, less than 5% of both CD7^+^CD56^neg^CD16^+^ and CD7^+^CD56^+^CD16^+^ NK cells express CXCR3 (Figure 4E). By comparison, approximately 70% of CD7^+^CD56^bright^CD16^neg^ immature NK cells express CXCR3, and HIV-1 infection is associated with a significantly lower frequency of CXCR3^+^ CD7^+^CD56^bright^CD16^neg^ immature NK cells compared to healthy controls (Figure 4E). In healthy controls, CXCR3 is expressed by a significantly lower frequency of CD7^+^CD56^neg^CD16^+^ compared to CD7^+^CD56^+^CD16^+^ NK cells (Figure 4E). HIV-1 infection was associated with a significant decrease in the frequency of CXCR3^+^CD7^+^CD56^+^CD16^+^ NK cells (Figure 4E). Taken together, CD7^+^CD56^neg^CD16^+^ and CD7^+^CD56^+^CD16^+^ NK cells are mature NK cell subsets with at least a fraction being terminally differentiated CD57^+^ NK cells; however, HIV-1-infection significantly alters the differentiation of both NK cell subsets.

### HIV-1 infection has a significant impact on the function of both NK cell subsets

**Figure 5:**
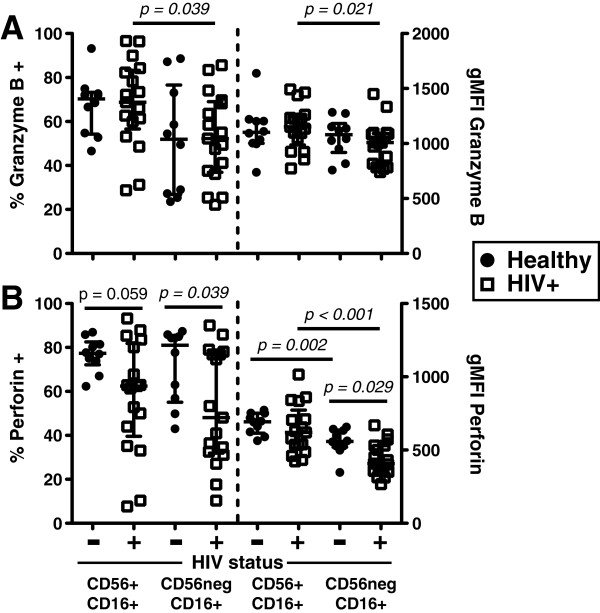
**Perforin and granzyme B expression in CD7**^**+**^**CD56**^**+**^**CD16**^**+ **^**and CD7**^**+**^**CD56**^**neg**^**CD16**^**+ **^**NK cells of healthy and chronically infected HIV-1 subjects. A**, Granzyme B expression in healthy donors and chronically infected HIV-1 subjects. **B**, Perforin expression in healthy donors and chronically infected HIV-1 subjects. Healthy subjects n = 10; chronic HIV-1-infected subjects n = 17. The median and 25th and 75th percentile are indicated on each graph. ( = healthy;  = HIV-1-infected).

Granzyme B and perforin are important effector molecules expressed by mature NK cells. In healthy controls, no statistically significant differences in the frequency of NK cells expressing granzyme B or the amount of granzyme B in the NK cell subsets was observed, although a trend toward a decreased frequency of CD7^+^CD56^neg^CD16^+^ NK cells expressing granzyme B was observed (Figure 5A). In contrast, the frequency of granzyme B-positive NK cells and the amount of granzyme B expression were significantly lower in CD7^+^CD56^neg^CD16^+^ NK cells of HIV-1-chronically-infected subjects. No statistically significant differences were observed between healthy control and HIV-1-chronically-infected subjects in granzyme B expression within the CD7^+^CD56^+^CD16^+^ or CD7^+^CD56^neg^CD16^+^ NK cell subsets (Figure 5A). A significantly lower amount of perforin was observed in CD7^+^CD56^neg^CD16^+^ NK cells of both healthy controls and HIV-1-chronically-infected subjects compared with CD7^+^CD56^+^CD16^+^ NK cells (Figure 5B). The frequency of cells expressing perforin in both CD7^+^CD56^+^CD16^+^ and CD7^+^CD56^neg^CD16^+^ NK cell populations was significantly lower in HIV-1-chronically-infected subjects. Furthermore, the amount of perforin in CD7^+^CD56^neg^CD16^+^ NK cells was significantly lower compared to CD7^+^CD56^+^CD16^+^ NK cells in HIV-1-chronically-infected subjects (Figure 5B). Taken together, significantly reduced granzyme B and perforin expression in CD7^+^CD56^neg^CD16^+^ NK cells of HIV-1-chronically-infected subjects might indicate this NK cell subset has recently degranulated in response to an encounter with target cells.

**Figure 6:**
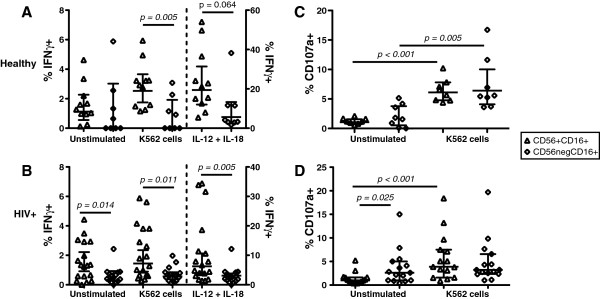
**Assessment of IFN and CD107a expression by CD7**^**+**^**CD56**^**+**^**CD16**^**+ **^**and CD7**^**+**^**CD56**^**neg**^**CD16**^**+ **^**NK cells following *in vitro *stimulation.** A-B IFNγ expression was compared between CD7^+^CD56^+^CD16^+^ and CD7^+^CD56^neg^CD16^+^ NK cells in **(A)** healthy and **(B)** chronic HIV-1-infected donors following incubation in media alone, IL-12 plus IL-18, or K562 target cells. C-D, CD107a expression was compared between CD7^+^CD56^+^CD16^+^ and CD7^+^CD56^neg^CD16^+^ NK cells in **(C)** healthy and **(D)** chronic HIV-1-infected donors following incubation in media alone or K562 target cells. A total of 10 healthy and 19 chronic HIV-1-infected donors were assessed. A minimum of 200 cells and 70 cells were analyzed for IFNγ and CD107 expression in CD7^+^CD56^+^CD16^+^ and CD7^+^CD56^neg^CD16^+^ NK cells, respectively, of healthy donors. A minimum of 550 cells and 100 cells were analyzed for IFNγ and CD107 expression in CD7^+^CD56^+^CD16^+^ and CD7^+^CD56^neg^CD16^+^ NK cells, respectively, of HIV-1-infected donors. The median and 25th and 75th percentile are indicated on each graph. ( = CD7^+^CD56^+^CD16^+^ NK cells;  = CD7^+^CD56^neg^CD16^+^ NK cells).

CD56^neg^CD16^+^ NK cells have been described as anergic and having poor cytokine and cytotoxic function *in vitro*[9,10,12]. Indeed, in both healthy controls and HIV-1-infected subjects, CD7^+^CD56^neg^CD16^+^ NK cells expressed significantly less IFNγ compared to CD7^+^CD56^+^CD16^+^ NK cells following K562 target cell stimulation (Figure 6A-B). IL-12 plus IL-18 stimulation did induce IFNγ secretion from CD7^+^CD56^neg^CD16^+^ NK cells in healthy controls, albeit less than CD7^+^CD56^+^CD16^+^ NK cells (Figure 6A). CD7^+^CD56^+^CD16^+^ NK cells from HIV-1-chronically-infected subjects had a significantly lower frequency of IFNγ-secreting cells in response to either K652 target cells (p = 0.033) or IL-12 plus IL-18 stimulation (p = 0.011) (Figure 6A-B). CD7^+^CD56^neg^CD16^+^ NK cells from HIV-1-chronically-infected subjects had a significantly higher frequency of IFNγ^+^ cells (p = 0.013) compared to healthy controls in the unstimulated condition. IFNγ responses induced by K562 target cells in CD7^+^CD56^neg^CD16^+^ NK cells from healthy and HIV-1-chronically-infected subjects were negligible. CD7^+^CD56^neg^CD16^+^ NK cells from HIV-1-chronically-infected subjects failed to produce IFNγ following cytokine stimulation (Figure 6B). In contrast to IFNγ responses, no significant differences in the frequencies of CD107a-expressing CD7^+^CD56^+^CD16^+^ and CD7^+^CD56^neg^CD16^+^ NK cells were observed following K562 target stimulation in healthy controls. Indeed, both subsets of NK cells were capable of degranulating in response to target cell stimulation (Figure 6C). In HIV-1-chronically-infected subjects, CD7^+^CD56^neg^CD16^+^ NK cells had a significantly higher frequency of CD107a expression in the unstimulated condition (Figure 6D). In contrast to their CD7^+^CD56^+^CD16^+^ NK cell counterpart, CD7^+^CD56^neg^CD16^+^ NK cells from HIV-1-chronically-infected subjects did not significantly degranulate following K562 stimulation (Figure 6C-D). A comparison of CD107a^+^ cells in CD7^+^CD56^+^CD16^+^ and CD7^+^CD56^neg^CD16^+^ NK cells in healthy and HIV-1-chronically-infected subjects did not result in any statistically significant differences. Taken together, CD7^+^CD56^neg^CD16^+^ NK cells from healthy controls were capable of degranulating following target cell stimulation. However, HIV-1 infection resulted in a significant defect in IFNγ secretion in both NK cell subsets and in degranulation in CD7^+^CD56^neg^CD16^+^ NK cells compared to CD7^+^CD56^+^CD16^+^ NK cells. Interestingly, HIV-1 infection also resulted in a higher frequency of IFNγ secretion and degranulation by CD7^+^CD56^neg^CD16^+^ NK cells in unstimulated conditions.

### Increased CD95 expression by CD7^+^CD56^neg^CD16^+^ NK cells

**Figure 7:**
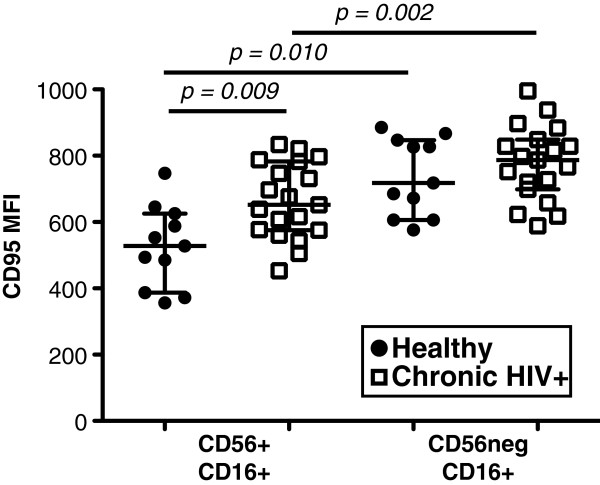
**Assessment of NK cell activation in healthy and chronically HIV-1-infected subjects.** CD95 expression on CD7^+^CD56^neg^CD16^+^ compared to CD7^+^CD56^+^CD16^+^ NK cells in healthy (n = 11) and chronically HIV-1-infected subjects (n = 19). The median and 25th and 75th percentile are indicated.

Granzyme B and perforin expression are lower in unstimulated CD7^+^CD56^neg^CD16^+^ NK cells compared to CD7^+^CD56^+^CD16^+^ NK cells (Figure 5). Furthermore, unstimulated CD7^+^CD56^neg^CD16^+^ NK cells have significantly higher basal CD107a expression compared to CD7^+^CD56^+^CD16^+^ NK cells, particularly in HIV-1-infected subjects (Figure 6). These data suggest that CD7^+^CD56^neg^CD16^+^ NK cells might represent an activated subset of NK cells that have recently engaged a target. Previous studies have shown that resting NK cells express a basal level of the apoptosis-inducing receptor CD95 on their cell surface and that upon activation either through Fc receptor [35] or cytokine stimulation [36], CD95 is significantly upregulated on NK cells. Kottilil *et al.* recently demonstrated that CD95 expression is increased on NK cells in HIV-1-infected subjects predominantly within the CD56^+^CD16^+^ NK cell subset [37]. Indeed, we observed that CD95 is significantly increased on both subsets of NK cells in HIV-1-chronically-infected subjects compared to healthy controls, but is particularly increased on CD7^+^CD56^neg^CD16^+^ NK cells in both healthy controls and HIV-1-chronically-infected subjects (Figure 7). These data suggest NK cells are activated during HIV-1 infection and that CD7^+^CD56^neg^CD16^+^ NK cells might represent NK cells that have recently engaged a target cell.

## Discussion

The lineage and biological role of CD3^neg^CD56^neg^CD16^+^ cells has remained poorly understood. The lack of a truly NK cell-specific marker and changes in cell phenotype during HIV-1 infection, as well as the potential overlap of phenotypic and functional properties of NK cells with other immune cell subsets, creates difficulties in studying rare and non-classical NK cell subsets. In humans, NK cells are most commonly identified as CD3^neg^CD56^+^ lymphocytes with functionally distinct subsets being further defined by CD16 expression. However, CD16 is expressed on many other immune cells, including granulocytes and a subset of dendritic cells (DCs), designated slanDCs [28]. Importantly, slanDCs do not express CD56; however, they do express CD13, CD33, HLA-DR, and high surface expression of CD16, which is the phenotype we observed on the CD7^neg^CD56^neg^ population of cells. Therefore, caution is needed in interpreting the frequencies and functions of CD56^neg^ NK cells. Here, we built upon our previous work using CD7 as an informative marker of NK cells and demonstrated that CD3^neg^CD56^neg^CD16^+^ cells are a mixed population of CD7^+^ NK cells and CD7^neg^ myeloid cells present in both healthy and HIV-1-infected subjects. Previously published microarray analyses support this observation as the CD3^neg^CD56^neg^CD16^+^ population exhibited a transcriptome more closely related to myeloid cells than traditional CD3^neg^CD56^+^CD16^+^ NK cells [25]. Our study has provided a more detailed understanding of the phenotype, function, and possible origin during chronic HIV-1 infection, of CD7^+^CD56^neg^CD16^+^ NK cells in healthy and HIV-1-infected subjects.

Little is known regarding the phenotype and function of CD56^neg^CD16^+^ cells in healthy subjects and whether these cells are similar to those observed in chronic viral infections. In HIV-1-negative subjects, we observed minor alterations in KIR, NCR, or C-type lectin-like receptors between the two NK cell subsets. In contrast to a previous report indicating a reduced density of NKp30 on CD56^neg^CD16^+^ cells in healthy donors [38], we observed that CD7^+^CD56^neg^CD16^+^ NK cells actually had increased NKp30 expression. Functionally, CD7^+^CD56^neg^CD16^+^ NK cells had impaired cytokine responses following K562 target cell or IL-12 plus IL-18 stimulation; however, they degranulated (as measured by CD107a-expression) similar to CD7^+^CD56^+^CD16^+^ NK cells following K562 target cell stimulation. By removing CD7^neg^ non-NK cells, we demonstrated that CD7^+^CD56^neg^CD16^+^ NK cells do not have as significantly altered of a phenotype as previously shown. While our results confirm that CD56^neg^ NK cells do exist in healthy donors, using CD7 provided for a precise determination of the frequency of CD56^neg^ NK cells present in the blood of healthy donors and allowed for analyses of their phenotype and functional characteristics.

Numerous studies have found decreases in both frequency and absolute numbers of NK cells defined as CD3^neg^CD56^+^ with a concomitant increase in a population of CD3^neg^CD56^neg^CD16^+^ cells in HIV-1 infection [6-8]. Our results confirm the expansion of a CD56^neg^ NK cell population; however, this population is not homogenous (<70% were CD7^+^CD56^neg^CD16^+^ NK cells). KIR expression was not significantly altered on either CD7^+^CD56^+^ or CD7^+^CD56^neg^ NK cells during HIV-1 infection compared to healthy donors. While HIV-1 infection was not associated with significant alterations in the frequency of cells expressing NKp30 or NKp46 in the CD7^+^CD56^+^ NK cell population, we did observe significant reductions in density of these receptors on CD7^+^CD56^neg^ NK cells. During HIV-1 infection, CD7^+^CD56^neg^ NK cells appeared to be mature; however, there were fewer CD57^+^ terminally differentiated CD7^+^CD56^neg^ compared to CD7^+^CD56^+^ NK cells. We recently demonstrated that CD57 is a marker of terminally differentiated NK cells [21], and that CD57^+^NKG2C^hi^ NK cells take weeks to develop *in vivo* following cytomegalovirus infection [22]. One possible explanation for the expansion of this CD7+CD56neg NK cell subset is that during HIV-1 infection, the already rapid turnover of NK cells [20] is further accelerated, preventing this subset of NK cells from terminally differentiating and gaining CD57 expression. Furthermore, our observation that CD7^+^CD56^neg^ NK cells express more of the apoptosis-inducing receptor CD95 further suggests these cells might undergo apoptosis prior to becoming terminally differentiated.

An alternative hypothesis is that the expanded population of CD7^+^CD56^neg^CD16^+^ NK cells during HIV-1 infection represents mature NK cells that arise at least in part when CD7^+^CD56^+^CD16^+^ NK cells engage target cells. NK cells are capable of killing multiple target cells and this results in a reduced, but never complete, loss of perforin and granzyme B [39]. Here, decreased granzyme B and perforin expression and increased expression of CD107a in the absence of *ex vivo* stimulation within the CD7^+^CD56^neg^CD16^+^ NK cells, particularly of HIV-1-infected subjects, suggests that CD7^+^CD56^neg^CD16^+^ NK cells may have recently engaged target cells *in vivo*. This is further supported by the increased expression of CD95 on CD7^+^CD56^neg^CD16^+^ NK cells, indicating these cells are more activated than their CD7^+^CD56^+^CD16^+^ NK cell counterparts.

Importantly, the frequency of CD7^+^CD56^neg^CD16^+^ NK cells decreases *in vivo* during antiretroviral treatment [9,11,40] and *in vitro* with the addition of IL-2 [12]. Indeed, IL-2 has been shown to help recover perforin and granzyme B expression in NK cells that have killed target cells [39]. Furthermore, one study suggests that IL-2 may be beneficial in restoring CD56^+^CD16^+^ NK cells as they observed an increase in CD56^+^CD16^+^ NK cells and a reduction in CD56^neg^CD16^+^ NK cells *in vivo*[41]. The *in vivo* efficacy of IL-2 plus antiretroviral therapy requires further exploration. Another interesting approach is the combinatorial use of statins, particularly hydrophilic statins, with cytokine therapies. Statins are known to protect against oxidative stress [42] and hydrophilic statins do not inhibit NK cell cytotoxicity [43]. Such therapies may afford an individual increased control of viral replication prior to initiating antiretroviral therapy and provide increased clearance of viral reservoirs during antiretroviral therapy or in the event of drug resistance. Future studies investigating the effects of cytokine and statin therapies in chronic inflammatory diseases are needed to determine whether they help maintain a healthy NK cell repertoire.

## Conclusion

Taken together, CD56^neg^CD16^+^ NK cells are a mixed population of CD7^+^ NK cells and CD7^neg^ myeloid cells present at a low frequency in healthy donors irrespective of CMV serology status and expanded in HIV-1 viremic subjects. Our results indicate CD7^+^CD56^neg^CD16^+^ NK cells are activated, mature NK cells that may have recently engaged target cells. Further *in vivo* studies are needed to evaluate treatments for maintaining NK cell functionality in HIV-1 viremic subjects through the use of cytokine and statin therapies.

## Methods

### Human subjects

Density gradient centrifugation n over Ficoll-Paque (GE Healthcare) was used to obtain PBMC from leukocyte concentrates of healthy volunteers (Stanford Blood Center). Additionally, PBMC were obtained from participants in two San Francisco-based HIV-1-infected cohorts, OPTIONS that recruits subjects during early HIV-1 infection [44] and SCOPE that recruits subjects during chronic HIV-1 infection [45]. Table 1 summarizes the subjects used from each HIV-1 cohort and their respective CD4^+^ T cell counts and viral loads. All persons gave informed consent to participate in this study, and the University of California, San Francisco Committee on Human Research approved this study.

### Phenotypic and functional characterization of cell subsets

With the exception of the following antibodies, all fluorophore-labeled antibodies used for phenotypic analysis have been previously described [23]: phycoerythrin (PE)-conjugated anti-Siglec 7 (BioLegend) and PE-conjugated anti-LIR-1 (Beckman Coulter). All stains were performed in the presence of 100 μg/mL human IgG to block Fc receptors. No significant differences in isotype-matched control Ig staining were observed between CD7^+^CD56^+^CD16^+^ NK cells and CD7^+^CD56^neg^CD16^+^ NK cells. Cells were analyzed by flow cytometry with a four-laser LSR-II instrument (BD Biosciences, San Jose, CA) as previously described [23] and data analyses were carried out using FlowJo flow cytometric analysis software version 9.3.1 (Tree Star, Ashland, OR).

### NK cell stimulation and intracellular cytokine staining

NK cell stimulation with K562 target cells or IL-12 plus IL-18, as well as intracellular IFNγ staining, was performed as previously described [23].

### Statistical analysis

Statistical analyses were performed with GraphPad Prism software (GraphPad Software). The nonparametric Mann–Whitney *U* test was used to compare between-group distributions with a significance threshold set at *p* < 0.05.

## Abbreviations

NK cell: Natural killer cell; HIV-1: Human immunodeficiency virus-1; DC: Dendritic cell; PBMC: Peripheral blood mononuclear cells.

## Competing interests

The authors declare that they have no competing interests.

## Authors’ contributions

JMM designed and performed experiments, analyzed results, made figures, and wrote the manuscript. SL-V. designed and performed experiments and assisted in writing the manuscript. VAY performed experiments and analyzed results. SGD, FMH, and JNM provided reagents and critical revision of the manuscript. LLL and DFN. participated in study design, discussion of the results, and provided critical editing of the manuscript. All authors read and approved the final manuscript.

## Supplementary Material

Additional file 1 **Gating strategy to identify NK cell subsets in a representative healthy donor.** Single, live cells were gated on lymphocytes based on forward and side scatter parameters. CD3^neg^, CD14^neg^, and CD19^neg^ cells were gated and used to identify classically defined NK cells using CD56 and CD16 expression. To eliminate any potential contaminating myeloid cells, CD7 was assessed on each subset of classically defined NK cells (CD56^br^CD16^neg^ (green), CD56^dim^CD16^+^ (red) and CD56^neg^CD16^+^ (teal)). All three subsets contained CD7^neg^ cells; however, the CD56^neg^CD16^+^ subset contained the highest proportion of CD7^neg^ non-NK cells. Overlaying the CD7^neg^ non-NK cells onto the CD7^+^ NK cells indicates the high overlap within the subsets and the usefulness of CD7 as an additional marker of NK cells. Click here for file

Additional file 2 **CD7 gating allows precise identification of NK cells.** CD56^+^CD16^+^ (red) or CD56^neg^CD16^+^ (blue) cell subsets were assessed for NKp30 or granzyme B expression without gating on CD7^+^ cells (left panels) or after gating on CD7^+^ NK cell subsets (right panels). Click here for file

Additional file 3 **Comparison of NK cell gating strategies on NKp30 and NKp46 expression.** (A) NKp30 and (B) NKp46 expression were assessed on NK cells defined in three ways; (1) CD7^+^CD56^+^CD16^+^ NK cells, (2) total CD7^+^CD56^+^ NK cells inclusive of CD56^bright^CD16^neg^, CD56^dim^CD16^neg^ and CD56^dim^CD16^pos^ NK cells and (3) CD7^+^CD56^neg^CD16^+^ NK cells. Click here for file

## References

[B1] Caligiuri MA Human natural killer cells Blood 2008 10 461 469 10.1182/blood-2007-09-077438 18650461 PMC2481557

[B2] Lanier LL Up on the tightrope: natural killer cell activation and inhibition Nat Immunol 2008 10 495 502 10.1038/ni1581 18425106 PMC2669298

[B3] Moretta A Bottino C Mingari MC Biassoni R Moretta L What is a natural killer cell? Nat Immunol 2002 10 6 8 10.1038/ni0102-6 11753399

[B4] Walzer T Blery M Chaix J Fuseri N Chasson L Robbins SH Jaeger S Andre P Gauthier L Daniel L Identification, activation, and selective in vivo ablation of mouse NK cells via NKp46 Proc Natl Acad Sci USA 2007 10 3384 3389 10.1073/pnas.0609692104 17360655 PMC1805551

[B5] Lanier LL Le AM Civin CI Loken MR Phillips JH The relationship of CD16 (Leu-11) and Leu-19 (NKH-1) antigen expression on human peripheral blood NK cells and cytotoxic T lymphocytes J Immunol 1986 10 4480 4486 3086432

[B6] Mansour I Doinel C Rouger P CD16+ NK cells decrease in all stages of HIV infection through a selective depletion of the CD16+CD8+CD3- subset AIDS Res Hum Retroviruses 1990 10 1451 1457 10.1089/aid.1990.6.1451 2150319

[B7] Voiculescu C Avramescu C Balasoiu M Turculeanu A Radu E Changes of blood CD16/CD56 (NK) and HLA-DR/CD3-positive lymphocyte amounts in HIV-infected children, as related to clinical progression and p24-antigen/p24-antibody presence FEMS Immunol Med Microbiol 1994 10 217 221 7529081 10.1111/j.1574-695X.1994.tb00496.x

[B8] Hu PF Hultin LE Hultin P Hausner MA Hirji K Jewett A Bonavida B Detels R Giorgi JV Natural killer cell immunodeficiency in HIV disease is manifest by profoundly decreased numbers of CD16 + CD56+ cells and expansion of a population of CD16dimCD56- cells with low lytic activity J Acquir Immune Defic Syndr Hum Retroviro 1995 10 331 340 7552495

[B9] Alter G Teigen N Davis BT Addo MM Suscovich TJ Waring MT Streeck H Johnston MN Staller KD Zaman MT Sequential deregulation of NK cell subset distribution and function starting in acute HIV-1 infection Blood 2005 10 3366 3369 10.1182/blood-2005-03-1100 16002429

[B10] Alter G Suscovich TJ Kleyman M Teigen N Streeck H Zaman MT Meier A Altfeld M Low perforin and elevated SHIP-1 expression is associated with functional anergy of natural killer cells in chronic HIV-1 infection AIDS 2006 10 1549 1551 10.1097/01.aids.0000237371.31315.48 16847410

[B11] Brunetta E Fogli M Varchetta S Bozzo L Hudspeth KL Marcenaro E Moretta A Mavilio D The decreased expression of Siglec-7 represents an early marker of dysfunctional natural killer-cell subsets associated with high levels of HIV-1 viremia Blood 2009 10 3822 3830 10.1182/blood-2009-06-226332 19710502 PMC2773483

[B12] Mavilio D Lombardo G Benjamin J Kim D Follman D Marcenaro E O’Shea MA Kinter A Kovacs C Moretta A Fauci AS Characterization of CD56-/CD16+ natural killer (NK) cells: a highly dysfunctional NK subset expanded in HIV-infected viremic individuals Proc Natl Acad Sci USA 2005 10 2886 2891 10.1073/pnas.0409872102 15699323 PMC549494

[B13] Sondergaard SR Ullum H Pedersen BK Proliferative and cytotoxic capabilities of CD16 + CD56- and CD16+/−CD56+ natural killer cells APMIS 2000 10 831 837 10.1111/j.1600-0463.2000.tb00006.x 11252817

[B14] Vieillard V Fausther-Bovendo H Samri A Debre P Specific phenotypic and functional features of natural killer cells from HIV-infected long-term nonprogressors and HIV controllers J Acquir Immune Defic Syndr 2010 10 564 573 20147841 10.1097/QAI.0b013e3181d0c5b4

[B15] Bjorkstrom NK Lindgren T Stoltz M Fauriat C Braun M Evander M Michaelsson J Malmberg KJ Klingstrom J Ahlm C Ljunggren HG Rapid expansion and long-term persistence of elevated NK cell numbers in humans infected with hantavirus J Exp Med 2011 10 13 21 10.1084/jem.20100762 21173105 PMC3023129

[B16] Gonzalez VD Falconer K Bjorkstrom NK Blom KG Weiland O Ljunggren HG Alaeus A Sandberg JK Expansion of functionally skewed CD56-negative NK cells in chronic hepatitis C virus infection: correlation with outcome of pegylated IFN-alpha and ribavirin treatment J Immunol 2009 10 6612 6618 10.4049/jimmunol.0901437 19846870

[B17] Nguyen S Morel V Le Garff-Tavernier M Bolgert F Leblond V Debre P Vieillard V Persistence of CD16+/CD56-/2B4+ natural killer cells: a highly dysfunctional NK subset expanded in ocular myasthenia gravis J Neuroimmunol 2006 10 117 125 10.1016/j.jneuroim.2006.05.028 16904757

[B18] Antonioli CM Airo P Dermatomyositis associated with lymphoproliferative disorder of NK cells and occult small cell lung carcinoma Clin Rheumatol 2004 10 239 241 10.1007/s10067-003-0814-2 15168153

[B19] Ginaldi L De Martinis M Monti D Franceschi C Chronic antigenic load and apoptosis in immunosenescence Trends Immunol 2005 10 79 84 10.1016/j.it.2004.11.005 15668122

[B20] Lutz CT Karapetyan A Al-Attar A Shelton BJ Holt KJ Tucker JH Presnell SR Human NK cells proliferate and die in vivo more rapidly than T cells in healthy young and elderly adults J Immunol 2011 10 4590–4598 21402893 10.4049/jimmunol.1002732 PMC3071442

[B21] Lopez-Verges S Milush JM Pandey S York VA Arakawa-Hoyt J Pircher H Norris PJ Nixon DF Lanier LL CD57 defines a functionally distinct population of mature NK cells in the human CD56dimCD16+ NK-cell subset Blood 2010 10 3865 3874 10.1182/blood-2010-04-282301 20733159 PMC2981540

[B22] Lopez-Verges S Milush JM Schwartz BS Pando MJ Jarjoura J York VA Houchins JP Miller S Kang SM Norris PJ Expansion of a unique CD57NKG2Chi natural killer cell subset during acute human cytomegalovirus infection Proc Natl Acad Sci USA 2011 10 14725 14732 10.1073/pnas.1110900108 21825173 PMC3169160

[B23] Milush JM Long BR Snyder-Cappione JE AJr C York VA Ndhlovu LC Lanier LL Michaelsson JL Nixon DF Functionally distinct subsets of human NK cells and monocyte/DC-like cells identified by co-expression of CD56, CD7, and CD4 Blood 2009 10 4823 4831 10.1182/blood-2009-04-216374 19805616 PMC2786291

[B24] Gonzalez VD Falconer K Michaelsson J Moll M Reichard O Alaeus A Sandberg JK Expansion of CD56- NK cells in chronic HCV/HIV-1 co-infection: reversion by antiviral treatment with pegylated IFNalpha and ribavirin Clin Immunol 2008 10 46 56 10.1016/j.clim.2008.03.521 18495540

[B25] Novershtern N Subramanian A Lawton LN Mak RH Haining WN McConkey ME Habib N Yosef N Chang CY Shay T Densely interconnected transcriptional circuits control cell states in human hematopoiesis Cell 2011 10 296 309 10.1016/j.cell.2011.01.004 21241896 PMC3049864

[B26] De Maria A Fogli M Costa P Murdaca G Puppo F Mavilio D Moretta A Moretta L The impaired NK cell cytolytic function in viremic HIV-1 infection is associated with a reduced surface expression of natural cytotoxicity receptors (NKp46, NKp30 and NKp44) Eur J Immunol 2003 10 2410 2418 10.1002/eji.200324141 12938217

[B27] Bigley V Spence LE Collin M Connecting the dots: monocyte/DC and NK subsets in human peripheral blood Blood 2010 10 2859 2860 10.1182/blood-2010-05-285676 20947692

[B28] Schakel K Kannagi R Kniep B Goto Y Mitsuoka C Zwirner J Soruri A von Kietzell M Rieber E 6-Sulfo LacNAc, a novel carbohydrate modification of PSGL-1, defines an inflammatory type of human dendritic cells Immunity 2002 10 289 301 10.1016/S1074-7613(02)00393-X 12354382

[B29] Guma M Angulo A Vilches C Gomez-Lozano N Malats N Lopez-Botet M Imprint of human cytomegalovirus infection on the NK cell receptor repertoire Blood 2004 10 3664 3671 10.1182/blood-2004-05-2058 15304389

[B30] Guma M Cabrera C Erkizia I Bofill M Clotet B Ruiz L Lopez-Bote M Human cytomegalovirus infection is associated with increased proportions of NK cells that express the CD94/NKG2C receptor in aviremic HIV-1-positive patients J Infect Dis 2006 10 38 41 10.1086/504719 16741880

[B31] Naeger DM Martin JN Sinclair E Hunt PW Bangsberg DR Hecht F Hsue P McCune JM Deeks SG Cytomegalovirus-specific T cells persist at very high levels during long-term antiretroviral treatment of HIV disease PLoS One 2010 10 e8886 10.1371/journal.pone.0008886 20126452 PMC2813282

[B32] Beziat V Descours B Parizot C Debre P Vieillard V NK cell terminal differentiation: correlated stepwise decrease of NKG2A and acquisition of KIRs PLoS One 2010 10 e11966 10.1371/journal.pone.0011966 20700504 PMC2917352

[B33] Campbell JJ Qin S Unutmaz D Soler D Murphy KE Hodge MR Wu L Butche EC Unique subpopulations of CD56+ NK and NK-T peripheral blood lymphocytes identified by chemokine receptor expression repertoire J Immuno 2001 10 6477 6482 10.4049/jimmunol.166.11.6477 11359797

[B34] Frey M Packianathan NB Fehniger TA Ross ME Wang WC Stewart CC Caligiuri MA Evans SS Differential expression and function of L-selectin on CD56bright and CD56dim natural killer cell subsets J Immunol 1998 10 400 408 9647249

[B35] Eischen CM Schilling JD Lynch DH Krammer PH Leibson PJ Fc receptor-induced expression of Fas ligand on activated NK cells facilitates cell-mediated cytotoxicity and subsequent autocrine NK cell apoptosis J Immunol 1996 10 2693 2699 8609385

[B36] Medvedev AE Johnsen AC Haux J Steinkjer B Egeberg K Lynch DH Sundan A Espevik T Regulation of Fas and Fas-ligand expression in NK cells by cytokines and the involvement of Fas-ligand in NK/LAK cell-mediated cytotoxicity Cytokine 1997 10 397 404 10.1006/cyto.1996.0181 9199873

[B37] Kottilil S Jackson JO Reitano KN O’Shea MA Roby G Lloyd M Yang J Hallahan CW Rehm CA Arthos J Innate immunity in HIV infection: enhanced susceptibility to CD95-mediated natural killer cell death and turnover induced by HIV viremia J Acquir Immune Defic Syndr 2007 10 151 159 10.1097/QAI.0b013e3180dc9909 17558334

[B38] Eller MA Eller LA Ouma BJ Thelian D Gonzalez VD Guwatudde D McCutchan FE Marovich MA Michael NL de Souza MS Elevated natural killer cell activity despite altered functional and phenotypic profile in Ugandans with HIV-1 clade A or clade D infection J Acquir Immune Defic Syndr 2009 10 380 389 10.1097/QAI.0b013e3181aa256e 19487954

[B39] Bhat R Watzl C Serial killing of tumor cells by human natural killer cells--enhancement by therapeutic antibodies PLoS One 2007 10 e326 10.1371/journal.pone.0000326 17389917 PMC1828617

[B40] Barker E Martinson J Brooks C Landay A Deeks S Dysfunctional natural killer cells, in vivo, are governed by HIV viremia regardless of whether the infected individual is on antiretroviral therapy AIDS 2007 10 17 2363 2365 10.1097/QAD.0b013e3282f1d658 18090295

[B41] Michaelsson J Long BR Loo CP Lanier LL Spotts G Hecht FM Nixon DF Immune reconstitution of CD56(dim) NK cells in individuals with primary HIV-1 infection treated with interleukin-2 J Infect Dis 2008 10 117 125 10.1086/524141 18171294 PMC4061976

[B42] Norata GD Pirillo A Catapano AL Statins and oxidative stress during atherogenesis J Cardiovasc Risk 2003 10 181 189 10.1097/00043798-200306000-00005 12775950

[B43] Raemer PC Kohl K Watzl C Statins inhibit NK-cell cytotoxicity by interfering with LFA-1-mediated conjugate formation Eur J Immunol 2009 10 1456 1465 10.1002/eji.200838863 19424968

[B44] Hecht FM Busch MP Rawal B Webb M Rosenberg E Swanson M Chesney M Anderson J Levy J Kahn JO Use of laboratory tests and clinical symptoms for identification of primary HIV infection AIDS 2002 10 1119 1129 10.1097/00002030-200205240-00005 12004270

[B45] Hunt PW Martin JN Sinclair E Bredt B Hagos E Lampiris H Deeks SG T cell activation is associated with lower CD4+ T cell gains in human immunodeficiency virus-infected patients with sustained viral suppression during antiretroviral therapy J Infect Dis 2003 10 1534 1543 10.1086/374786 12721933

