# Supplementary figures and images for: CD56negCD16+ NK cells are activated mature NK cells with impaired effector function during HIV-1 infection
Source: Retrovirology. 2013 Dec 18;10:158. doi: 10.1186/1742-4690-10-158 (PMC3892122; doi:10.1186/1742-4690-10-158)

# Additional File 1

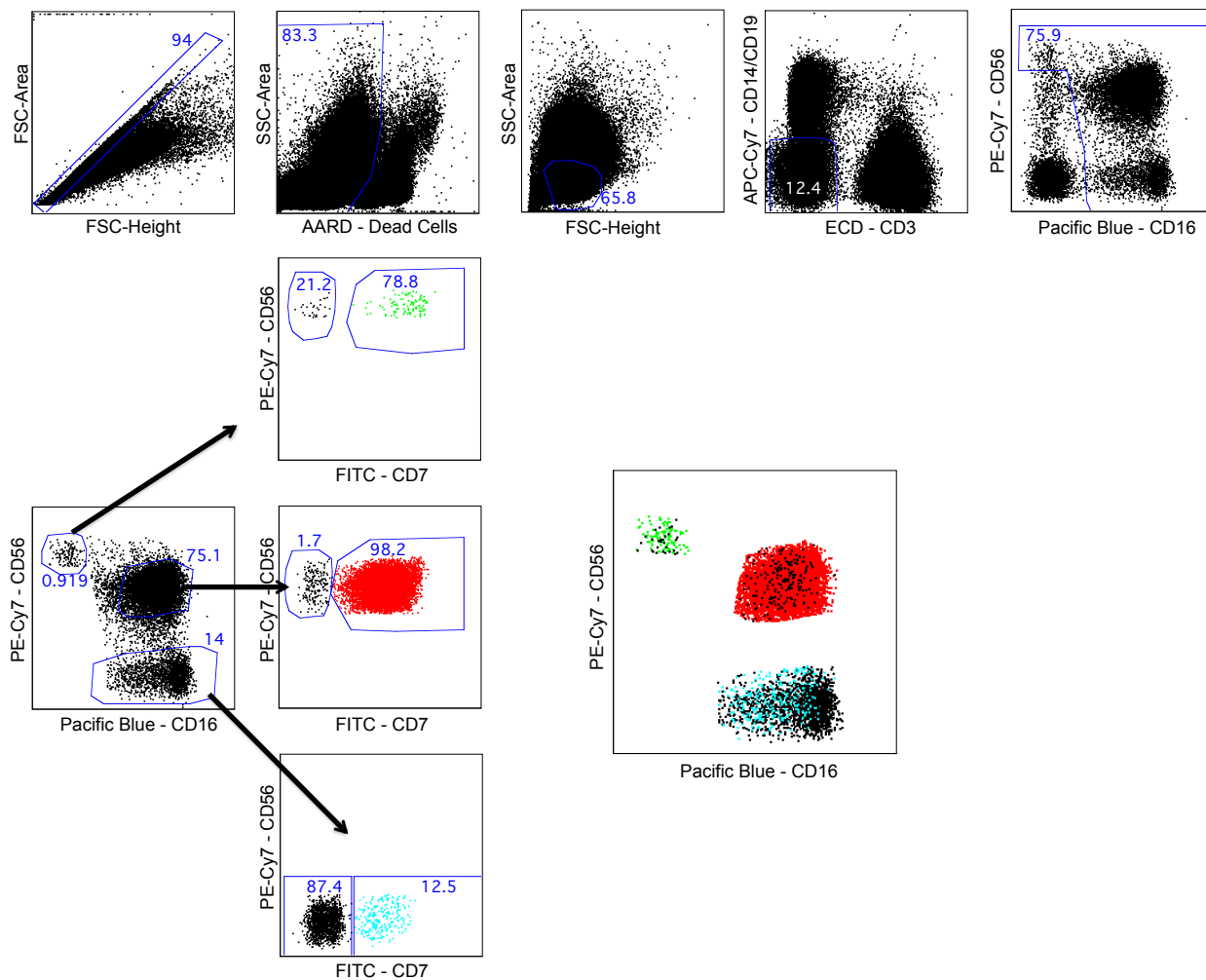

Supplement: Additional file 1 — Gating strategy to identify NK cell subsets in a representative healthy donor. Single, live cells were gated on lymphocytes based on forward and side scatter parameters. CD3neg, CD14neg, and CD19neg cells were gated and used to identify classically defined NK cells using CD56 and CD16 expression. To eliminate any potential contaminating myeloid cells, CD7 was assessed on each subset of classically defined NK cells (CD56brCD16neg (green), CD56dimCD16+ (red) and CD56negCD16+ (teal)). All three subsets contained CD7neg cells; however, the CD56negCD16+ subset contained the highest proportion of CD7neg non-NK cells. Overlaying the CD7neg non-NK cells onto the CD7+ NK cells indicates the high overlap within the subsets and the usefulness of CD7 as an additional marker of NK cells. [file 1742-4690-10-158-S1.pdf]

## Additional File 2

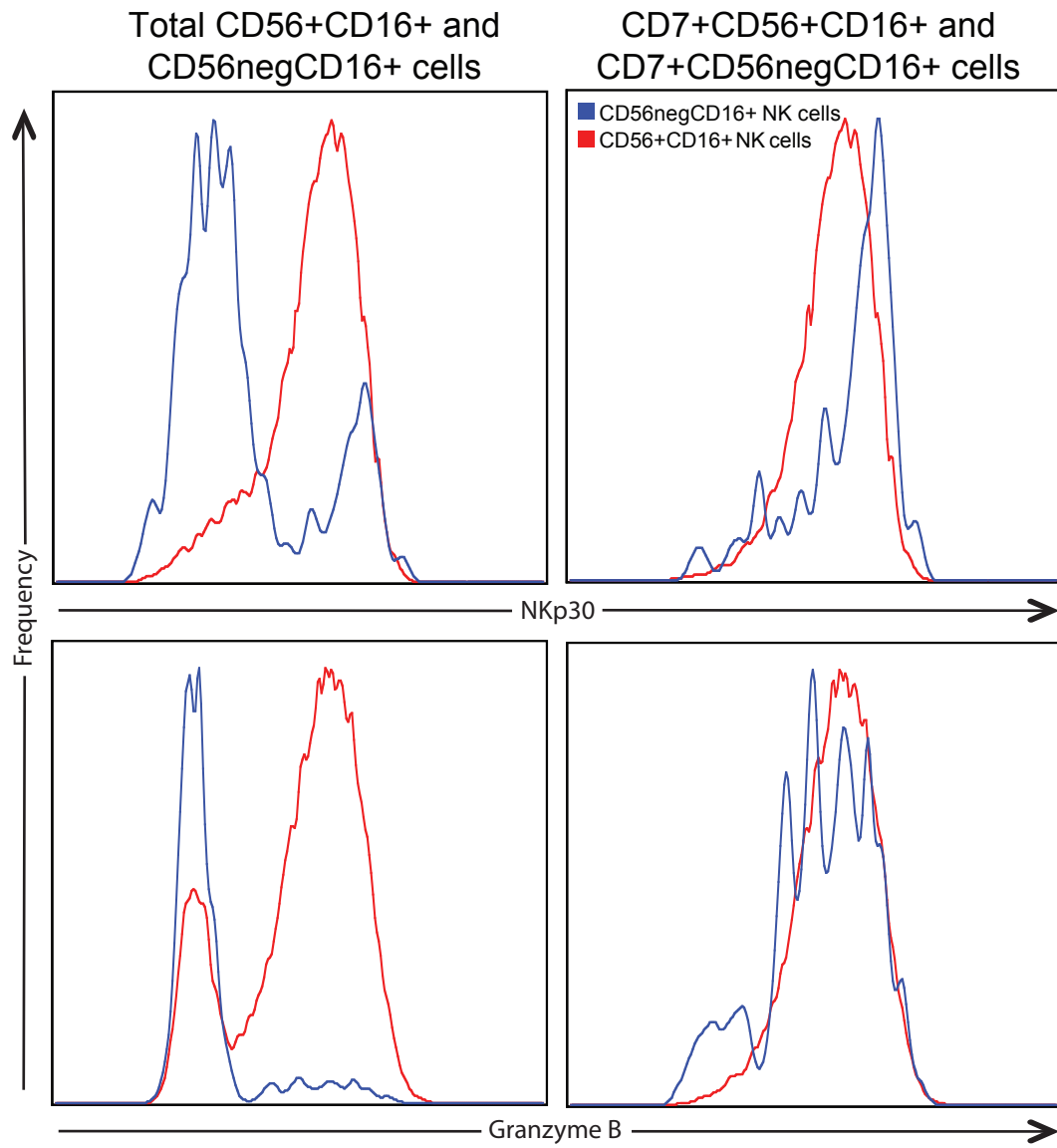

Supplement: Additional file 2 — CD7 gating allows precise identification of NK cells. CD56+CD16+ (red) or CD56negCD16+ (blue) cell subsets were assessed for NKp30 or granzyme B expression without gating on CD7+ cells (left panels) or after gating on CD7+ NK cell subsets (right panels). [file 1742-4690-10-158-S2.pdf]

# Additional File 3

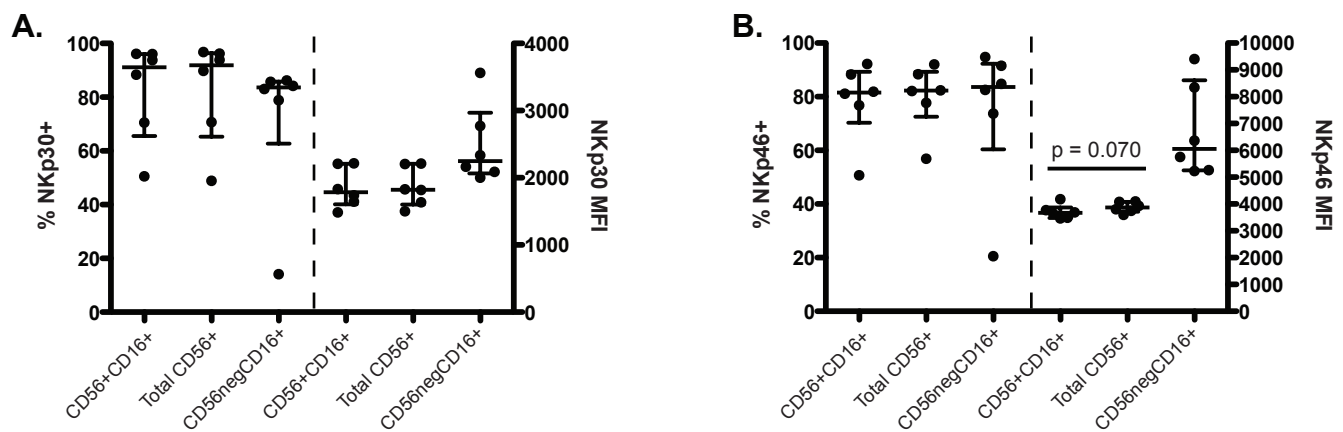

Supplement: Additional file 3 — Comparison of NK cell gating strategies on NKp30 and NKp46 expression. (A) NKp30 and (B) NKp46 expression were assessed on NK cells defined in three ways; (1) CD7+CD56+CD16+ NK cells, (2) total CD7+CD56+ NK cells inclusive of CD56brightCD16neg, CD56dimCD16neg and CD56dimCD16pos NK cells and (3) CD7+CD56negCD16+ NK cells. [file 1742-4690-10-158-S3.pdf]
